# Community-Based Designed Pilot Cooking and Texting Intervention on Health-Related Quality of Life among College Students

**DOI:** 10.3390/ijerph21030293

**Published:** 2024-03-02

**Authors:** Makenzie Barr-Porter, Amelia Sullivan, Emma Watras, Caitlyn Winn, Jade McNamara

**Affiliations:** 1 Department of Dietetics and Human Nutrition, University of Kentucky, Lexington, KY 40514, USA; makenzie.barr@uky.edu; 2 School of Food and Agriculture, University of Maine, Orono, ME 04469, USA; amelia.sullivan@maine.edu (A.S.); emma.watras@maine.edu (E.W.); caitlyn.winn@maine.edu (C.W.)

**Keywords:** nutrition assessment, college, food security, mental health, diet quality, emerging adults

## Abstract

As emerging adulthood is an important area of life for developing healthful habits, the current study aimed to test the feasibility of a pilot program for improving health-related quality of life (HRQOL), food security, and diet quality among college students. Students 18–26 years old were recruited from two land-grant institutions to participate in an online survey and self-selected to participate in an intervention study. An intervention program was developed by student peers to include (1) a four-session cooking curriculum and a (2) semester-long text message program to share health resources on the relevant college campus. Diet quality, food security, and mentally healthy days were assessed. Baseline to post-program changes were assessed using paired *t*-tests. Cohen’s d was used to determine effect size estimates. In the full sample (N = 65), “days/month when mental health was not good” and “days/month feeling worried, tense, or anxious” significantly improved (*p* < 0.05). Dietary quality measures of total short Healthy Eating Index (sHEI) score and total vegetable intake significantly improved throughout the intervention. Subsamples of (1) food-insecure participants (n = 22) and those with (2) dietary quality below the 50th percentile (n = 29) both had positive improvements following the program. Health promotion programs should be formed, adapted, or expanded in an effort to improve health among our next generation of workers. College and university environments should focus on mental health, diet, and food security among their constituents, particularly with regard to those at risk.

## 1. Introduction

Young adults’ (ages 18–35) lifestyle habits and behaviors are inscribed from their youth environment and parental influence. As they leave their childhood homes and attend college, many young adults experience newfound living independence. Paralleling these changes are deviations in diet, activity, stress, and other lifestyle situations and behaviors. Due to several confounding factors acting in a small window of one’s lifespan, young adults are known to have unhealthy lifestyles and the highest prevalence of mental health disorders among the age groups [1].

The transition to college is typically associated with unfamiliar levels of pressure and responsibility and consequently poorer mental health. In 2021, the prevalence of any mental health illness (AMI) was found to be the highest in young adults (33.7%) when compared to younger and older age groups [2]. Impaired mental health not only contributes to poor quality of life but also impacts physical abilities, resulting in a greater risk of developing chronic disease [3]. A major contributor to poor mental health status among college students is stress and dealing with fatigue and anxiety due to the stressors of university academic and social life [4]. While a vast majority of students report having access to services that help them with their physical and mental health, adverse effects on mental status are still prevalent [5]. Furthermore, young adults face difficulties post-graduation, such as struggles with functioning at work and work absences, if mental health issues are ignored [6].

Food insecurity likely contributes to the stress induced by college and subsequent poor mental health. Many undergraduates suffer from high rates of food insecurity. In fact, the rates of food insecurity among college students are double the rate for U.S households (35% to 42% versus 14%) [7,8]. Food insecurity has multifaceted effects on college students, adversely impacting their health-related quality of life. The literature reveals food-insecure students report significantly higher perceived stress; poor sleep quality; disordered eating behaviors [9]; consuming significantly fewer servings of fruits, vegetables, and legumes daily; and unfavorable academic experiences, such as trouble focusing in class, failing courses [10], and a low GPA [9]. Yet, college students often face the burden of food insecurity alone, leading to feelings of negative self-worth surrounding their food security statuses [11].

Food insecurity further exacerbates poor diet quality among college students. In a sample of over 1000 college students, 97% of participants did not meet fruit and vegetable recommendations and consumed 100 calories above the added sugar recommendation [12]. Studies specifically comparing food-secure to food-insecure college students consistently find that students with food insecurity report eating significantly fewer fruits and vegetables and more processed foods compared to their food-secure counterparts [10,13,14,15]. Additionally, food-insecure students unintentionally suffer from greater eating disturbances, as food-insecure students often report skipping meals entirely to afford academic-related expenses, such as rent and tuition [11]. Moreover, food-insecure students provide contradicting statements regarding their perception of a ‘normal college lifestyle’, with some perceiving food insecurity and unhealthy eating patterns as normal [10]. 

High rates of food insecurity paired with poor dietary habits, and health-related quality of life, put young adults at a greater risk for chronic disease [1,16]. Nevertheless, research has shown that with better diet quality, college students are less likely to report anxiety and depression, which underscores the importance of addressing modifiable risk factors associated with chronic disease and mental health [17]. Unfortunately, conflicting perceptions of food security status contribute to college students’ poor mental health, causing confusion, shame, stigma, and fear regarding food decisions. Shame and stigma often prevent students from exploiting external resources for acquiring healthy food to combat poor diet quality, such as SNAP benefits, or campus resources, such as food pantries [11]. Despite facing higher rates of food insecurity than other subpopulations [11], the determinants of food insecurity and interventions aimed at improving food security among college students are understudied. 

To improve the food security and health behaviors of young adults, programming needs to be culturally relevant. A way to improve the feasibility, sustainability, and buy-in of behavior change is to involve stakeholders in the process and progression of designing, implementing, and evaluating programs. Community-based participatory research (CBPR) applies core principles that, as explained by Simona and colleagues, “(a) foster joint ownership in the identification of health priorities, the development and evaluation of research strategies and their design, and the dissemination of findings; (b) a keen recognition and appreciation for the importance of stakeholder-driven priorities, research, and solutions; (c) building capacity of both stakeholders and researchers to engage in research collaboratively; and (d) recognizing that conducting the research is not the endpoint but continues on with a commitment to dissemination, spread, adoption and sustainability [4,18]”.

Researchers have conducted interventions within the college student population using CBPR, which led to improvements in diet quality and mealtime behavior regulation, physical activity, and alcohol consumption [19], but mental health and food security have not been explored. When looking for guidance on evidence-based programming to influence behavior change for these variables, theories of behavior change, such as Social Cognitive Theory (SCT), can be implemented. SCT highlights the importance of cognitive, behavioral, and environmental factors. Other community health interventions have proven feasible and successful at improving a multitude of behaviors among varying demographic groups when programming is developed utilizing methods such as cooking demonstrations, nutrition education [20,21], and text message campaigns [22,23]. 

The timing of young adulthood provides a key opportunity to enhance the health and longevity of our next generations of leaders and employees. Overall, young adults are at greater risk of developing AMIs, and, as a population with a history of poor diet quality and high rates of food insecurity, intervention strategies designed to improve health-related quality of life of young adults are warranted. The current research aimed to test the feasibility of a pilot health program, developed using CBPR, for improving the health-related quality of life (HRQOL), food security, and diet quality of college students. It was hypothesized that after participating in the program, college students would report an improvement in HRQOL, specifically in the mental health domain, food security, and diet quality.

## 2. Materials and Methods

### 2.1. Study Design

This study has a pre-post design, which allowed us to assess the feasibility of using a pilot wellness program, implemented over the course of a semester, to improve health-related quality of life, food security, and diet quality of college students. This study was conducted in accordance with the Declaration of Helsinki, and the protocol received expedited review and approval from both universities’ Institutional Review Boards, i.e., University of Kentucky Institutional Review Board (#61400; 18 May 2021) and the University of Maine (14 July 2020).

### 2.2. Participants and Recruitment

College students were recruited across campus from two land-grant institutions in January 2023. An electronic survey was distributed to full-time undergraduate students through email listservs. Inclusion criteria included being between the ages of 18 and 26 years, currently enrolled as an undergraduate student, and able to read and understand the English language. Survey completion was optional. Students were given incentives to participate in the survey, having the option to enter a raffle to win a USD 25 gift card. At the end of the online survey, students were given the option to enroll in the intervention program (involving a cooking class and text messaging). Informed consent was provided to each student that participated in the intervention before beginning the survey and then again to students before participating in the first in-person cooking class. Students were allowed to remove themselves from the program or data collection at any time. 

### 2.3. Intervention

The overarching study intervention used a community-based participatory research approach to develop a feasible and acceptable college-based program. Collaboratively, 7 undergraduate college student research partners participated in a 16-week college course in fall 2021 to assess their college environment for healthfulness and worked collaboratively across two Universities to develop a program that addressed the identified needs of their peers as a whole [24]. Details on this course, research student participation, and collaborative process for the program design were published previously [24]. Student research partners from both universities ultimately formed into an advisory team and met together at a 3-day workshop in January 2022 to decide on a cohesive program [24]. Intervention strategies were decided upon by employing CBPR methods, utilizing the nominal group technique, data from students’ community needs assessment, and each student’s knowledge as an attendant at each of the universities. Student advisory team included 7 undergraduate students who were a part of the fall 2021 course and 3 graduate research students. The wellness program that was ultimately developed through a collaborative effort by the student advisory team was titled *College Cooking Connection* and consisted of a four-class cooking/nutrition course and a semester-long (spanning approximately 16 weeks) text-messaging campaign. The student advisory team and researchers worked together throughout 2022 to finalize details of the program, such as the nutrition/cooking topics and recipes and the text messages, for implementation in 2023 [24].

The cooking course component of the intervention was designed by graduate nutrition students, with input from undergraduate students, and overseen by two registered dietitians. The cooking courses were then taught in the Spring 2023 semester by graduate and undergraduate nutrition students, who were all overseen by a registered dietitian. Each class lasted approximately 90 min and was broken up into education on a specific nutrition topic (e.g., MyPlate, mental health, and meal prep) and a cooking/eating component. Cooking classes were held on each respective campus at a site that included a teaching-kitchen-style room. Five sections of each class (i.e., five rounds of class 1, five rounds of class 2, etc.) were held on weekdays at each site to accommodate up to 15 participants per session. Classes were offered in flexible time frames to accommodate students’ demanding schedules. At the first class, participating students received reusable plastic utensils, Tupperware, and tote bags. Participants were additionally incentivized with a weekly Amazon e-gift card of increasing value for each class they participated in (class 1 = USD 10, class 2 = USD 15, class 3 = USD 20, and class 4 = USD 25) and excess food cooked during class. See Table 1 for an overview of the College Cooking Connection Curriculum.

The text message component of the intervention was used to share health resources and activities offered at each of the college campuses. In the development of the intervention, student research partners developed text message content for the intervention. Student research assistants were responsible for identifying health resources and events occurring throughout the semester and then disseminated that information to the participants. Example text messages include the following:

1. “February is American Heart month! The American Heart Association recommends 150 min of moderate-intensity aerobic activity or 75 min per week of vigorous aerobic activity to support heart health. Head down to the campus rec center to take part in a fitness class. Class sessions are linked here: LINK HERE”
2. “Did you know the counseling center offers free and confidential counseling sessions for students living in [state]? To schedule an initial consultation appointment, call the center at [contact information]. For more information visit: LINK HERE”
3. “This Wednesday March 1st kicks off nutrition month! Did you know [Campus Dietitian] offers free and confidential nutrition counseling to any student who holds a meal plan on campus? She is available by appointment [dates] in person or via Zoom. For more information and to set up your consultation visit: LINK HERE”
4. “Need to decompress? Check out the Spa located in room [Location on Campus]. The Spa offers programs to promote mindfulness and ease anxiety, as well as comfy seating, Yogibos, tea, activities, and even a Nintendo switch that can be used [dates/times]!”
5. “Looking to save money, support local businesses, and enjoy the freshest of ingredients? Check out the Farmer’s Market located [Location] every [date/time]! To check out the dates and calendar here: LINK HERE”
6. “As the semester comes to an end, budgeting money for groceries can be more of a challenge. Here’s a reminder that the [Name of Campus Food Pantry] is a campus food pantry, available to all students and staff members! Find the hours and location here: LINK HERE”

### 2.4. Instruments, Measures, Procedures, and Data Analysis

The pilot program was assessed using a pre-post design with a pre-post assessment to explore group effects. Electronic surveys using Qualtrics were distributed using college students’ email addresses at the beginning of the Spring 2023 semester and then again at the end of the Spring 2023 semester (approximately 16 weeks). We included validated survey items that assessed students’ HRQOL using the Center for Disease Control’s (CDC) Health Days Module [25], food security using the USDA Food Security Screener [26], diet quality using the Short Healthy Eating Index (sHEI) [27] total score, and food component scores, along with demographic variables. 

Food security status (FSS) was assessed using the 10-item USDA Household Food Security Screener [26], a scale validated and considered reliable to use in the adult population. Scores were calculated and summed. FSS scores of 0–2 were classified as high/marginal food security, and scores of 3–10 were classified as low food/very-low food security status. 

Items used to assess HRQOL were taken from the CDC’s Health Days Module [25]. These items included “for the past 30 days, about how many days did you feel sad, blue, or depressed?” and “for the past 30 days, about how many days did you feel worried, tense, or anxious?”, in which means and standard deviations are reported for days/months for the different measures of HRQOL. 

Overall diet quality was assessed utilizing the sHEI [27], and components of diet quality were assessed utilizing the sHEI subscales (i.e., fruit, vegetable, added sugar, sodium, and saturated fat.) The sHEI, a validated tool for assessing young adults, is a 22-item survey assessing 13 food groups. Based on self-reported food group consumption, a summative final percentage score between 0 and 100 is calculated, with higher scores indicating a healthier dietary quality intake in line with the Dietary Guidelines for Americans. The sHEI subscales reveal total cups of fruits, vegetables, dairy servings, added sugar in teaspoons, sodium in grams, and whole grains in ounce equivalents per day [27].

Only intervention-enrolled participants who completed the pre- and post-survey and attended at least one of the four classes were included in the analysis. Results were analyzed using SPSS software (IBM Corp. Released 2022. IBM SPSS Statistics for Macintosh, Version 29.0. Armonk, NY: IBM Corp). Descriptive statistics were used to identify sample characteristics. Participants’ demographic data were assessed using means and standard deviations for continuous variables and frequencies for categorical data. Responses to the Healthy Days Module, food security screener, and sHEI total and component scores were scored and averaged for continuous variables or reported as frequencies for categorical variables. A paired *t*-test was used to determine changes in continuous variables from baseline to after the study, and the McNemar test was used to assess differences in paired categorical variables from baseline to after the study [28]. Significance for both assessments was set at *p* < 0.05. For the paired *t*-tests, Cohen’s d was used to determine effect size estimates, categorized as 0.2 = small; 0.5 = medium; and 0.8 = large [29], and 95% confidence intervals (CI) were reported using the McNemar test.

Two subsamples of students participating in the intervention who reported (1) being food insecure and (2) had a sHEI score below the 50th percentile for assessing changes were also assessed. Students who were identified as being food insecure at baseline were examined using the paired McNemar x^2^ test with a 95% CI to identify if significant changes in food security status had occurred. Students who were identified as being below the 50th percentile for total HEI score at baseline were examined using paired *t*-tests to identify if significant changes were made in their total diet quality and component scores (fruit, vegetable, added sugars, sodium, and whole grains). Cohen’s d was used to determine effect size estimates, categorized as 0.2 = small; 0.5 = medium; and 0.8 = large [29].

Lastly, information about the texting component of the program was collected in the post survey. Similar to other texting interventions [22,23], students were asked four questions regarding their experience with the texting intervention to gauge the feasibility and appropriateness of the program. The questions included (1) “How valuable did you find the texts you received throughout the semester” (not valuable to very valuable), (2) “Did you attend any events or visit and of the resources discussed in the texts you received” (yes or no), (3) “Do you feel you are more aware of the resources available on your campus after receiving the texts” (yes or no), and (4) “Do you have any comments or suggestions for this program for future semesters.” Data for questions 1–3 were analyzed using frequencies, and direct quotes from the open-ended question were reported. 

## 3. Results

The baseline survey was emailed to 13,398 undergraduates, and 809 students responded. After removing those with missing or incomplete data, it was observed that 124 students indicated an interest in enrolling in the College Cooking Connection. Of those 124 students, 65 participated in one or more of the four cooking classes and completed pre-post assessments. The overall sample of participants in this study (N = 65) consisted of individuals who were predominantly female (71.2%), white (80.3%), identified as heterosexual (60.6%), were approximately 20 years in age, and rated their health as “good” (42.4%) (Table 2). Years in school were relatively equally spread across undergraduate rankings (freshman through senior).

From baseline to post-program (approximately 16 weeks), there was a significant improvement in “days/month when mental health was not good” (M = 11.57 ± 8.51 to M = 10.11 ± 8.38 days/month, *p* < 0.05) and “days/month feeling worried, tense, or anxious days/month” (M = 13.65 ± 9.81 to M = 11.71 ± 9.47 days/month, *p* < 0.05). The effect size for the difference between time for both measures was calculated using Cohen’s D, resulting in small to medium effects of 0.253 and 0.272, respectively. Significant changes were also reported for dietary quality measures of total sHEI score (M = 47.98 ± 9.83 to M = 49.93 ± 10.29, *p* < 0.05) and total vegetable intake across the intervention (M = 2.66 ± 0.75 to M = 2.85 ± 0.58, *p* < 0.05). The effect size for the difference between the baseline and post-intervention results for total sHEI and total vegetable component score resulted in Cohen’s D values of 0.248 and 0.287, respectively, both indicating small to medium effects. Food security status was not significantly different from baseline to post-intervention, with a McNemar x^2^ = 1.92, *p* = 0.17 (95% CI: −3.47, 18.85). See Table 3 for the results.

Additionally, a subsample of participants (n = 23, 36.9%) was identified as food insecure at baseline (corresponding to a food security score of 2 or higher). In this subsample, there was a significant change in food security status from baseline to post-intervention (McNemar x^2^ = 9.00, *p* < 0.01) (95% CI: 59.21,16.93). Of those who were food insecure at baseline (100%), almost 40% reported being food secure at the post-assessment timepoint (Table 4).

Diet quality changes from baseline to post-intervention were assessed for those students whose sHEI total scores were below the 50th percentile (n = 29; average score: 48.92). sHEI total score and component scores (fruit, vegetable, added sugar, sodium, and whole grains) were assessed for change and effect size. The variables total sHEI score, total fruits, and total vegetables all significantly improved in the subsample group (all *p*’s < 0.05), with small to medium effect sizes (Table 5).

A subsample of students (46%, n = 30) reported their experiences with the text message component of the program. Based on the results, 23.3% of the subsample reported the text messages to be valuable/very valuable, with the majority (63.3%) reporting that the text messages were “somewhat valuable”. Forty percent of people reported attending events or visiting resources shared through the text messages, and 83.3% reported being more aware of the resources available on their campus after receiving the texts. Open-ended feedback included linking PowerPoint slides from the nutrition lessons, highlighting a recipe of the week, including more information about food, sharing student fundraisers, and making the messages “less ominous”.

## 4. Discussion

The current study aimed to establish the feasibility of using the College Cooking Connection to improve health-related quality of life, food security status, and dietary quality among college student participants. Following the intervention, the participants’ days per month of perceived poor mental health, diet quality, and food security improved in a subsample of students who began the program at a time when they were food insecure. These results showcase that it is feasible to improve modifiable risk factors of chronic disease through a nutrition/cooking and texting program conducted on college campuses.

Research has captured the rise in and/or worsening of mental health challenges among young adults, particularly throughout the transition to college [30]. In our study, it was shown that mentally healthy days improved from baseline to post-program implementation among our full cohort. This is especially important considering the lasting effects of the coronavirus disease 2019 (COVID-19) pandemic and the impact it has had on college students’ mental health [31]. Research has shown that COVID-19 worsens college students’ mental health in various ways [32]; however, as the current study shows, college health programming and resources can the reduce risk of mental health disorders. Soria and Horgos [33] showed that during the pandemic, college students who reported that they had a greater sense of belonging and felt valued at their colleges were less likely to report symptoms of depression or anxiety. They also found that college students who reported food insecurity had increased odds of both depression and generalized anxiety [33]. These findings, taken together, underscore the importance of the college environment and understanding how it can be utilized to support the best health outcomes for this population.

Similarly, several studies on food insecurity have been conducted on young adults, particularly in the college setting, sharing similar sentiments of unhealthy life consequences indicating the need for policies, interventions, and programming to alleviate the burden. Unfortunately, there are limited studies concerning the effect of evidence-based programming on reducing food insecurity among young adults. Of the few studies conducted, Torrey and colleagues utilized a “Food Scholarship Program” in which students received bi-monthly pre-packed food totes with preparation directions and recipe cards. As a result, student vegetable HEI scores significantly improved, and significant micronutrient intake improvements were observed, but the prevalence of food insecurity (low or very low) at baseline did not significantly change post-intervention [34]. In contrast, Ahmed and colleagues ran a student-led intervention, “Playing with Our Food”, in which student leaders shared food research and played educational games with participants to raise awareness of and reduce stigma regarding food insecurity and related campus programs. Although this study did not directly assess the frequency changes in food insecurity among participants, it did reveal that students felt more inclined to utilize campus food programs (i.e., campus food pantries) and felt open to changes related to food and food programming due to the intervention [34].

Not only does food insecurity impact mental health, but it also impacts nutritional status. In an analysis of EAT 2010–2018 data, Larson and colleagues identified nutrition-related behaviors more likely to be displayed among young adult individuals who were screened and assessed as being food insecure, including regarding poor diet quality, limited healthy foods at home, skipping breakfast, consumption of fast food, and binge eating [35]. Historically, dietary patterns among young adults have included higher amounts of fats and processed foods along with limited intake of fruits and vegetables [36], whole grains, and low-fat dairy. Despite some previous trends in dietary quality indicating improvements in component scores (i.e., greater high-quality carbohydrate intake) [37], there continues to be a lower quality overall for over half of youth and adolescents [38] supporting the need for dietary and behavioral-based interventions among younger populations.

Among a subsample group of students in the current study who had dietary quality scores below the 50th percentile, total dietary quality, total fruit score, and total vegetable score all significantly improved following the intervention. Like food security status changes, though sHEI scores improved, this improvement still maintained a dietary quality scoring below 50%, indicating that dietary intake was still less than recommended. These findings are similar to others indicating young adults have the worst dietary quality among the age groups [39].

Improvements in fruit and vegetable consumption are noteworthy for this population. As food insecurity is known to influence related conditions such as obesity and related chronic conditions, a modest increase in fruit and vegetable consumption is clinically meaningful. A previous meta-analysis of prospective cohort studies indicated all-cause mortality and cardiovascular risk decreased with an increase in servings of fruit and vegetables [40]. A similar meta-analysis of young adult dietary interventions identified that there was a higher proportion of studies that did not find significant changes in dietary quality after interventions with these populations. Though the authors suggest that this age group may be associated with the greatest difficulty in changing dietary habits, in our full cohort, we did find that the intervention was successful in significantly changing dietary quality [39].

As mentioned, the transition to a university or college setting can generate a strong shift in eating patterns [39] and preparations [41]. Consumption of meals and beverages on a college campus has been associated with worse dietary quality scores [42]. Inversely, home meal preparation and overall cooking skills have been associated with greater dietary quality and have been suggested to be incorporated into intervention programming for this population [43]. Intervention studies on dietary quality changes among young adult college students have also indicated positive outcomes on mental health and give evidence that a healthy diet can lessen symptoms of depression [44].

### 4.1. Limitations

Our study is not without limitations. The student participants self-selected to participate through university-wide recruitment and were not randomly selected. Therefore, the individuals who participated in this study may have ultimately been interested in nutrition, well-being, and health. Additionally, due to the nature of an electronic survey and the assessment of behavioral items, inherent bias should additionally be noted due to the nature of the self-reported data. Specifically, regarding the HRQOL variables, while they captured the participants’ perceptions of days of poor mental health, they did not capture what was contributing to poor mental health. In future studies on this population, researchers may want to include a more robust measure of mental health such as the Warwick–Edinburgh Mental Wellbeing Scale [45]. Likewise, generalizability to other college and university populations is limited, as our cohort was a small, homogeneous sample drawn from two university communities and lacked a control comparison group. While our demographics are not representative of the larger population of the United States or the overall demographics of college students; they are representative of the demographics of the two universities where the study was conducted [46]. Future research should include a larger sample and a control group to deduce if the improvements observed were due to the intervention.

As we intended to implement minimal inclusion criteria to obtain feedback from a wide audience, this may have led to a lack of information collected from those most at risk of structural vulnerabilities. Due to our purposive sampling, we are unable to determine external validity, and our study may not have robust representation. Likewise, due to the nature of this program, namely, including two intervention components (cooking classes and text messaging), we are unable to differentiate the intervention component impacts individually. In future implementations of this program, researchers could consider recruiting separate cohorts for each intervention component to identify which approaches elicit a favorable response. Finally, as these data were collected as part of a larger study, future work should consider more robust mixed-methods questioning of food environmental influences.

### 4.2. Implications for Research and Practice

This study both supports previous findings in the literature and provides further insight into at-risk groups of young adults. Despite its limitations, the current study captured the unique and positive outcomes of a college health promotion intervention. These findings provide further insight into the power of health-related interventions for young adults, particularly among those with poor diet quality or food insecurity. Future studies may consider a longer intervention program with intentional recruitment efforts toward including students who are at risk for poor dietary intake (i.e., lower dietary quality scores or screening for food insecurity).

Since research shows that students are better protected against poor mental health when feeling supported and valued by their university, providing programming for students that cultivates a sense of belonging may be particularly beneficial, especially for groups at greater risk of poor mental health (i.e., females, food-insecure students, and sexual minority students). This is especially important post-COVID-19 and -social distancing, which resulted in feelings of isolation and increased stress/anxiety. The most effective programs for improving health and promoting belonging on college campuses will be ones designed in partnership with students.

Efforts for expanding reach to all college students, rather than a self-selected few, may be found through nutrition and well-being programs or opportunities embedded within college curricula. Previous work has utilized core courses that are part of each student’s basic degree hours to implement well-being classes. Providing a full-term (i.e., semester or quarter) college course promoting healthful lifestyle behaviors may be a beneficial avenue for combining the components of evidence-based interventions. In the meta-analysis by Ashton and colleagues, the effectiveness of Behavior Change Techniques was assessed based on the Behavior Change Techniques Taxonomy [47]. The authors found that some of the more effective intervention techniques included (1) habit formation, (2) assessing the salience of consequences, (3) adding objects to the environment, and (4) action planning [39].

With improving food security rates among emerging adults, several position pieces have been published regarding the advancement of food security status by several pioneers in the field. Expanding our knowledge of food security rates and risks, assessing college environments, adapting or developing programs, implementing change, and assessing the impact of these programs [48,49], in addition to the expansion of governmentally funded programs for this widely left-out group, further research, and support for health equity across campuses and universities and for marginalized students, particularly via taking an individualized approach per campus [49], would greatly benefit this field.

## 5. Conclusions

Following a pilot intervention conducted on a college campus aimed at improving health-related quality of life for emerging adults, the participants’ mental health improved, along with their diet quality. Food security status did not change for the full sample, though in a subsample group of food-insecure students, students who reported low food security at baseline significantly improved their food security statuses following the intervention. Likewise, among individuals below the 50th percentile for diet quality at baseline, their diet quality significantly improved. Generalizability should be considered when expanding these results to other emerging adult populations.

Continued approaches should be formed, adapted, or expanded in an effort to improve health among our next generation of workers. College and university environments should continue to focus on mental health, diet, and food security among their constituents, particularly for those at risk. Providing knowledge and learning experiences for these individuals should not come without the care of their health, well-being, and longevity.

## Figures and Tables

**Table 1:** Overview of the College Cooking Connection Curriculum.

Lesson No.	Lesson Topic
1	MyPlate, parts of the knife, knife skills
2	Gut–brain connection, nutrients for supporting mental health
3	Budgeting, tips for saving money at the grocery store
4	Meal planning

**Table 2:** Characteristics of participants (N = 65).

Variable	Total Participants
Age (years), mean (SD)	20.00 (1.4)
Sex, female, n (%)	47 (71.2)
Race/ethnicity, n (%)	
White	53 (80.3)
African American	2 (3.0)
Hispanic or Latino	1 (1.5)
Native American	8 (12.1)
Other	2 (3.0)
Sexual Identity, n (%)	
Heterosexual	40 (60.6)
Homosexual	5 (7.6)
Bisexual	13 (19.7)
Other	3 (4.5)
Year in School, n (%)	
Freshman	18 (27.3)
Sophomore	15 (22.7)
Junior	17 (25.8)
Senior	16 (24.2)
General Health Status, n (%)	
Excellent	4 (6.1)
Very good	22 (33.3)
Good	28 (42.4)
Fair	10 (15.2)
Poor	2 (3.0)

**Table 3:** Program outcomes for health-related quality of life, food security, and diet quality (N = 65).

Variable	Baseline	Post-intervention	*p*-Value	Cohen’s D
Mental health not good (days/month), mean (SD)	11.57 (8.51)	10.11 (8.38)	0.024 *	0.253
Sad, Blue, or Depressed (days/month), mean (SD)	8.52 (7.79)	7.86 (8.22)	0.213	0.101
Worried, Tense, or Anxious (days/month), mean (SD)	13.65 (9.81)	11.71 (9.47)	0.018 *	0.272
Very Health and Full of Energy (days/month), mean (SD)	9.86 (8.11)	10.31 (8.32)	0.304	0.064
Total sHEI (SD)	47.98 (9.83)	49.93 (10.29)	0.033 *	0.248
Total fruit (SD)	3.33 (1.42)	3.99 (1.51)	0.062	0.21
Total vegetable (SD)	2.66 (.75)	2.85 (.58)	0.016 *	0.287
Added Sugar (SD)	3.36 (3.30)	3.10 (3.22)	0.227	0.078
Sodium (SD)	3.57 (2.02)	3.64 (1.97)	0.404	0.032
Whole grains (SD)	3.59 (2.40)	3.85 (2.40)	0.166	0.13
Food security score (n, (%))				
High or marginal	41 (63.1)	46 (70.8)	0.17	
Low and very low	24 (36.9)	19 (29.2)		

* Significance set at *p* < 0.05. Cohen d’s Effect size estimate categorizes: 0.2 = small; 0.5 = medium; 0.8 = large. Health-related quality-of-life items were assessed using the CDC’s Healthy Days Module. Healthy Eating Index total score and component scores were assessed using the Short Healthy Eating Index (sHEI). Food Security Scores was assessed using the USDA’s 10-item food security screener. High or marginal food security includes scores of 0–2, and low/very-low food security includes scores of 3–10.

**Table 4:** Changes in food security status for a subsample of food-insecure baseline participants (n = 23).

Timepoint	Food Secure	Food Insecure	*p*-Value
Baseline (n (%))	0 (0%)	23 (100%)	<0.01
Post (n(%))	9 (39.1%)	14 (60.9%)	

Food Security Score was assessed using the USDA’s 10-item food security screener. Scores of 3 or higher indicate food insecurity. Food-secure status includes values of 0–2, and food-insecure status includes values of 3–10. McNemar chi-square test was used to assess changes in paired sample categorical data from baseline to post-intervention. Significance was set at *p* < 0.05.

**Table 5:** Subsample of those below the 50th percentile for diet quality (n = 29).

Variable	Baseline	Post	*p*-Value	Cohen’s D
Total sHEI	40.6 (6.7)	44.6 (9.8)	0.012 *	0.444
Total fruits	2.8 (1.5)	3.5 (1.7)	0.034 *	0.351
Total vegetables	2.2 (.74)	2.6 (.64)	0.011 *	0.452
Added Sugar	2.24 (3.1)	1.9 (2.8)	0.301	0.098
Sodium	3.2 (2.15)	3.2 (2.04)	0.483	0.008
Whole Grains	2.7 (2.3)	3.1 (2.2)	0.136	0.209

* Significance set at *p* < 0.05. Cohen d’s Effect size estimates are categorized as 0.2 = small; 0.5 = medium; 0.8 = large. Healthy Eating Index total score and component scores were assessed using the Short Healthy Eating Index (sHEI).

## Data Availability

Data are available upon reasonable request made to the authors.
